# Avocado consumption and cognitive health: Refining expectations for single-food dietary interventions

**DOI:** 10.1016/j.jnha.2026.100864

**Published:** 2026-05-08

**Authors:** Mònica Bulló

**Affiliations:** a Nutrition and Metabolic Health Research Group (NuMeH), Department of Biochemistry and Biotechnology, Rovira i Virgili University (URV), Reus, Spain; b Institut de Recerca Biomèdica Catalunya Sud (IRBCatSud), Reus, Spain; c Center of Environmental, Food and Toxicological Technology – TecnATox, Rovira i Virgili University, Reus, Spain; d CIBER Physiology of Obesity and Nutrition (CIBEROBN), Carlos III Health Institute, 28029 Madrid, Spain

Diet plays a central role in shaping trajectories of cognitive aging, with growing interest in identifying specific foods that may preserve brain health before the onset of clinical impairment. In this context, the randomized controlled trial by Lee and colleagues provides timely and rigorous evidence examining whether daily consumption of one avocado, without any other nutritional or lifestyle modifications, influences cognitive performance over six months in adults with central obesity.

Avocados are a nutrient-dense food rich in monounsaturated fatty acids, dietary fiber, and bioactive compounds such as lutein, zeaxanthin and polyphenols, all components that have been linked to vascular health, inflammation, cognitive function and brain health [[1], [2], [3]]. Despite this favorable nutritional profile, evidence from intervention studies specifically examining avocado consumption and cognition remains limited. Two previous clinical trials have investigated these effects, both observing limited and domain‑specific improvements, mainly in executive functioning. Of these, one study examined a range of cognitive domains in a healthy, normal‑weight adult population [4], whereas the other focused on adults with overweight or obesity but was limited to younger and middle‑aged participants, and evaluated only a single cognitive domain [5]. Given that obesity is associated with metabolic dysfunction, systemic inflammation, and an increased risk of cognitive impairment, extending this line of research to individuals with central obesity is particularly relevant [6]. The study by Lee et al., extends the previous works by employing a comprehensive neuropsychological battery spanning multiple cognitive domains, enrolling participants from young adulthood to late life, and implementing a longer intervention period than earlier avocado studies.

Despite the excellent dietary compliance and the strong methodological rigor, including an appropriate sample size, a well-designed intervention, validated nutritional and cognitive assessment tools, and a robust statistical approach, the study found no significant cognitive benefits attributable to avocado consumption across memory, processing speed, executive function, or reaction time. These null findings are important and should be interpreted within the broader context of the study design and the inherent constraints of single-food interventions. In particular, they help refine expectations regarding the magnitude and detectability of cognitive effects when an isolated dietary component is introduced over a relatively short duration and during midlife, a life stage characterized by gradual and often subtle cognitive change. Detecting meaningful differences in cognitive performance over six months may therefore be challenging, even in well-conducted and potentially adequate powered trials.

Rather than indicating a lack of nutritional relevance, these findings underscore the likelihood that measurable cognitive benefits may require sustained exposure periods and multidimensional lifestyle strategies integrating healthy dietary patterns, physical activity and sleep quality, rather than reliance on isolated food additions. Increasing evidence highlights that cognitive health is shaped by the dynamic interplay of behavioral, metabolic, and biological factors, suggesting that single-food interventions, even when nutritionally favorable, may be insufficient to meaningfully alter cognitive trajectories [7]. Consistent with this perspective, the results reported by Lee et al. align with evidence suggesting that dietary interventions tend to exert domain-specific and context-dependent effects, rather than producing broad improvements in global cognition.

Several features of the study design further contextualize these findings and strengthen their interpretation. By focusing on adults with central obesity, the authors addressed a metabolically vulnerable population at increased risk of future cognitive decline; however, obesity-related metabolic dysregulation and chronic low-grade inflammation may also attenuate responsiveness to nutritional interventions, particularly over relatively short intervention periods. The use of a comprehensive neuropsychological battery covering multiple cognitive domains is a key strength, enabling detection of domain-specific effects that might be missed when relying solely on global cognitive measures. At the same time, the lack of age-related differences suggests that consuming one avocado per day, without other lifestyle changes and within limited time period, is unlikely to meaningfully influence cognitive trajectories across adulthood. In addition, the modest intake of bioactive compounds provided by one avocado daily may be insufficient when compared with doses used in trials employing supplements, extracts, or nutraceutical formulations, potentially contributing to the lack of detectable cognitive effects.

Importantly, these results should not be interpreted as evidence against the role of diet in cognitive health, but rather as an indication that measurable cognitive benefits may require broader dietary pattern changes, longer or sustained interventions, higher cumulative exposure, or even targeting individuals at more advanced stages of cognitive or metabolic vulnerability, when changes may be more readily detectable.

Overall, this study contributes meaningfully to a growing body of research showing that the relationships between diet and brain health are complex and not always easy to detect over short periods, highlighting the challenges of translating biological plausible mechanisms into measurable cognitive outcomes. Importantly, the study shows why future research needs to consider metabolic health, baseline cognitive status, long-term dietary habits, and other lifestyle factors when evaluating the effects of specific foods. Well-designed studies reporting null results like this one are valuable because they help set realistic expectations and support clear, evidence-based public health messages. In sum, Lee et al. present a carefully conducted trial that advances our understanding of how single foods relate to cognitive aging, reinforcing that while avocados, while are a nutritionally valuable component of the diet, their addition without any other lifestyle modification over a six-months period is unlikely to produce measurable cognitive benefits in adults with central obesity.

## Declaration of Generative AI and AI-assisted technologies in the writing process

AI was used to review the English.

## Funding

M.B. received the ICREA Academy 2022 Distinction from the Autonomous Government of Catalonia. This work was supported by the Department of Research and Universities of the Government of Cataloniathrough the Nutrition and Metabolic Health Research Group (2021 SGR 00213).

## Declaration of competing interest

The authors declare that they have no known competing financial interests or personal relationships that could have appeared to influence the work reported in this paper.
